# Perceived stress among physician parents during COVID-19 pandemic

**DOI:** 10.1192/j.eurpsy.2022.1234

**Published:** 2022-09-01

**Authors:** N. Regaieg, D. Ben Touhemi, A. Fayala, J. Boudabous, W. Kammoun, K. Khemakhem, I. Hadj Kacem, H. Ayadi, Y. Moalla

**Affiliations:** Hedi Chaker University Hospital, Child And Adolescent Psychiatry, Sfax, Tunisia

**Keywords:** physician parents, Covid-19, Perceived stress

## Abstract

**Introduction:**

Stress among physician parents is still poorly studied, especially during the SARS-COV-2 pandemic.

**Objectives:**

To describe the stress of being both a doctor and a parent during COVID-19 epidemic.

**Methods:**

It was a cross-sectional, descriptive and analytical study, carried out on google drive in March 2021, and relating to 93 Tunisian medical parents. We used a questionnaire containing the parents’ personal and professional data as well as the perceived stress scale (PSS10).

**Results:**

The majority of parents (94.7%) were women. The average age was 34.43 years old. The average age of marriage was 29.6 years for men and 25.4 years for women. The majority of parents (89.4%) had one or two children and 70.2% were satisfied with their relationship with their children. On another side, 71.3% of doctors had to provide on duty services in the hospital, with 44.1% providing 3-4 on-calls per month, while 69% were providing on duty services in the COVID units. The average PSS score was 22.6. The distribution of scores indicated medium and high stress level in respectively 84.9% and 14% of parents. Furthermore, the PSS score was negatively correlated with the marriage age (p = 0.046, r = -0.2). On the other hand, no association was observed with the children number nor with the satisfaction of the relationship with his child.

**Conclusions:**

It follows from our study that stress among physician parents is at a fairly high level. Managing this stress during a pandemic is not easy and requires the activation of several defense mechanisms.

**Disclosure:**

No significant relationships.

